# Unveiling novel insights in acute myeloid leukemia through single-cell RNA sequencing

**DOI:** 10.3389/fonc.2024.1365330

**Published:** 2024-04-22

**Authors:** Jianbiao Zhou, Wee-Joo Chng

**Affiliations:** ^1^ Cancer Science Institute of Singapore, Center for Translational Medicine, National University of Singapore, Singapore, Singapore; ^2^ Department of Medicine, Yong Loo Lin School of Medicine, National University of Singapore, Singapore, Singapore; ^3^ NUS Center for Cancer Research, Center for Translational Medicine, Singapore, Singapore; ^4^ Department of Hematology-Oncology, National University Cancer Institute of Singapore (NCIS), The National University Health System (NUHS), Singapore, Singapore

**Keywords:** acute myeloid leukemia (AML), single cell RNA-sequencing (scRNA-seq), intratumoral heterogeneity, leukemia stem cell (LSC), bone marrow microenvironment, immunotherapy, novel therapy

## Abstract

Acute myeloid leukemia (AML) is a complex and heterogeneous group of aggressive hematopoietic stem cell disease. The presence of diverse and functionally distinct populations of leukemia cells within the same patient’s bone marrow or blood poses a significant challenge in diagnosing and treating AML. A substantial proportion of AML patients demonstrate resistance to induction chemotherapy and a grim prognosis upon relapse. The rapid advance in next generation sequencing technologies, such as single-cell RNA-sequencing (scRNA-seq), has revolutionized our understanding of AML pathogenesis by enabling high-resolution interrogation of the cellular heterogeneity in the AML ecosystem, and their transcriptional signatures at a single-cell level. New studies have successfully characterized the inextricably intertwined interactions among AML cells, immune cells and bone marrow microenvironment and their contributions to the AML development, therapeutic resistance and relapse. These findings have deepened and broadened our understanding the complexity and heterogeneity of AML, which are difficult to detect with bulk RNA-seq. This review encapsulates the burgeoning body of knowledge generated through scRNA-seq, providing the novel insights and discoveries it has unveiled in AML biology. Furthermore, we discuss the potential implications of scRNA-seq in therapeutic opportunities, focusing on immunotherapy. Finally, we highlight the current limitations and future direction of scRNA-seq in the field.

## Background

Acute myeloid leukemia (AML) is a complex and aggressive group of hematopoietic stem cell disorders. It is characterized with excessive accumulation of immature myeloid blasts in the bone marrow (BM) and peripheral blood (PB) (1–3). The uncontrolled proliferation is driven by genetic mutations that affect signaling pathways regulating cell cycle progression and apoptosis. Mutations in genes such as *FMS-like tyrosine kinase 3* (*FLT3*), *Nucleophosmin* (*NPM1*), and other regulators of cell growth contribute to the dysregulation of these processes. In AML, there is a disruption in the normal process of hematopoietic cell differentiation. Immature myeloid blasts, which are the precursor cells to mature blood cells, fail to undergo proper maturation (4, 5). Instead, they become arrested at an early stage of development. This differentiation block is often associated with specific genetic mutations, such as those in transcription factors like *CEBPA*, *RUNX1* or genetic alterations affecting epigenetic regulation, including mutations in *TET2*, *DNMT3A* or isocitrate dehydrogenase 1 (*IDH1*)/*IDH2* (4, 6, 7).

The standard therapy for AML consists of induction with 7 days of cytarabine plus 3 days of an anthracycline (e.g., daunorubicin or idarubicin), followed by consolidation with additional chemotherapy or stem-cell transplantation (8, 9). This “7 + 3” regimen has yielded a 5-year survival of 20% to 35% in young patients and 10% in older patients (10–12). During the last 10 years, progress in the field has resulted in the creation of targeted agents tailored for specific mutations (1, 13). Many of these agents have obtained approval from the Food and Drug Administration and are under investigation in ongoing clinical trials (13–15). However, the effectiveness of these targeted drugs as monotherapies has been hindered by the development of drug resistance over time (16, 17). There are still subsets of patients with limited therapeutic options (18). Finding effective treatments for these groups remains a challenge. The presence of diverse and functionally distinct populations of leukemia cells within the same patient’s BM or PB poses a significant challenge in diagnosing and treating AML (19, 20). A significant portion of patients with AML exhibit resistance to chemotherapy or targeted therapies, leading to refractory or relapsed disease with a worse prognosis (14, 21, 22).

Since the emergence of next-generation Sequencing (NGS) in 2005, a multitude of comprehensive bulk RNA-seq studies have provided further insight into the pathogenesis, molecular classification, characterization of recurrent mutations, and detection of minimal residual disease in AML (7, 23, 24). However, bulk RNA-seq measures the average gene expression from all cells in the sample, but fails to distinguish between individual cells within a population and lacks the ability to distinguish individual cell variations (25, 26). In contrast, single cell (sc) RNA-sequencing (scRNA-seq) allows the analysis of gene expression at the resolution of individual cells, providing insights into cell-to-cell variability (27, 28). scRNA-seq also facilitates the exploration of individual cell dynamics, including cell differentiation trajectories, a crucial aspect of both normal hematopoiesis and leukemogenesis research (29–31). The application of scRNA-seq has revolutionized our understanding of AML by enabling the dissection of cellular hierarchies, identification of rare cell populations, as well as novel cell types. scRNA-seq empowers researchers to uncover the dynamic transcriptional profiles of individual leukemic cells (32). The characterization of subpopulations with distinct molecular profiles and cellular functions has not only broadened our understanding of disease heterogeneity but has also provided potential targets for more precise therapeutic interventions (33).

Adult AML has a genomic and epigenetic profile distinct from that of pediatric AML. Adult AML often exhibits a higher frequency of some DNA mutations mentioned above. However, mutations of F*LT3-ITD*, *NPM1* are less commonly found and mutations of *TP53* and *DNMT3A* are almost absent in pediatric AML (34). On other side, somatic structural variants are approximate 10 times higher in pediatric AML as compared to adult AML (34). Therefore, they could be considered as two different disease entities. Here, we summarize findings from various RNA-seq studies in adult AML that dissected the cellular and molecular heterogeneity of the AML ecosystem, including leukemia stem cells (LSCs), immune cells and their interactions with AML bone marrow microenvironment. This review delves into the recent advancements in understanding the novel mechanisms of drug resistance and relapse in AML. We also highlight the transformative potential of single-cell analysis in the development of novel and personalized treatment strategies with the focus on immunotherapy for fighting AML.

## Unmasking heterogeneity

**Figure 1:**
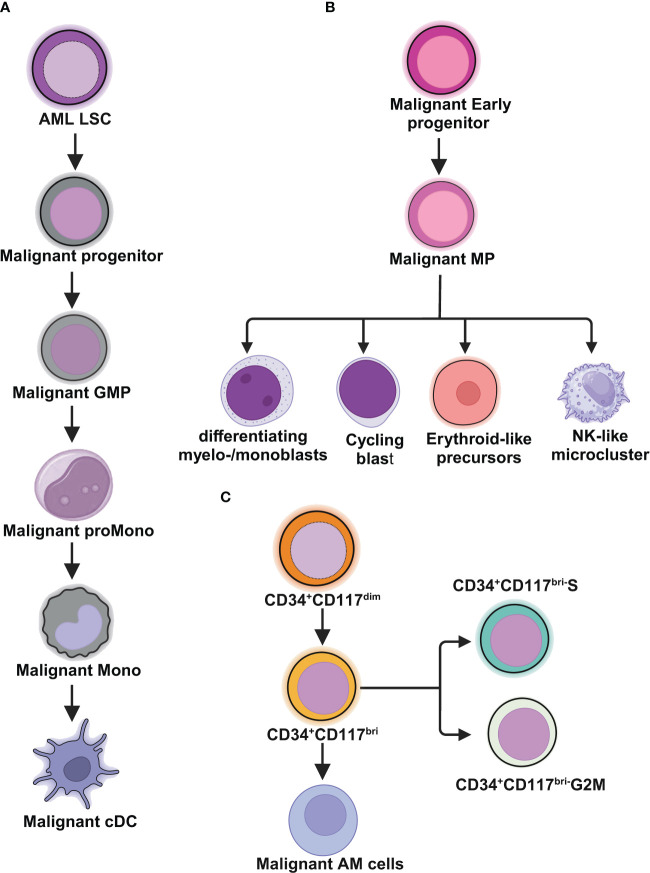
Schematic illustrations depict the leukemic spectrum at different stages of differentiation trajectory revealed by RNA-seq studies. **(A)** Unbiased single cell transcriptomic classifier identifies six types of AML cells mirroring the normal myeloid differentiation axis from hematopoietic stem cell (HSC) to myeloid (HSC-like, progenitor-like, granulocyte-monocyte progenitor (GMP)-like, promonocyte-like, monocyte-like, or conventional dendritic cell (cDC)-like malignant cells. The abundances of these 6 different stages of malignant cells vary significantly among AML patients. These abundances correlate closely with cell morphology and surface phenotypes. The scRNA-seq data reveal greater malignant cell diversity compared to flow cytometry-based estimates, highlighting the potential of scRNA-seq to provide more detailed information on AML cell types and differentiation states. **(B)** In *NPM1* ^mut^ AML, at the peak of differentiation trajectory, malignant early progenitors differentiate into malignant myeloid progenitor (MP). These MP cells give rise to various malignant progeny cells, including differentiating myelo-/monoblasts, actively cycling blasts, erythroid-like precursors, and a microcluster with NK-like characteristics. **(C)** In AML with t(8;21), by integrating clinical immunophenotypic characterization, the scRNA-seq analysis delineates five distinct intrapatient leukemic cell clusters. At the top of the hierarchy are CD34^+^CD117^dim^ cells, representing the earliest differentiation trajectory, followed by CD34^+^CD117^bri^ blasts and abnormal myeloid cells with partial maturation (AM) at the end of differentiation axis. The CD34^+^CD117^bri^ blasts can be further divided into CD34^+^CD117^bri^-S blasts and CD34^+^CD117^bri^-G2M blasts based on their cell cycle status inferred from their single cell transcriptomic profiles. This figure was created with BioRender.com.

**Figure 2:**
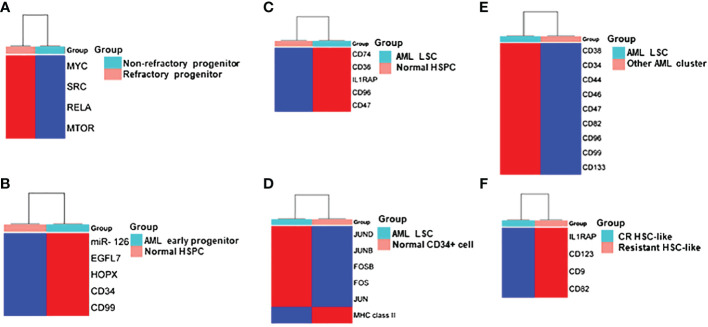
Heatmap Comparisons of differential gene expression determined by scRNA-seq in **(A)** refractory progenitor vs non-refractory progenitor from AML patients; **(B)** AML early progenitor vs normal HSPC; **(C)** AML LSC vs normal HSPC; **(D)** AML LSC vs normal CD34+ cell; **(E)** LSC vs other clusters from AML patients; **(F)** HSC-like cell from chemotherapy-resistant AML patients vs AML patients who achieved complete remission (CR). Red and blue shadings represent higher and lower relative expression levels, respectively. These heatmaps were constructed by using SRplot (https://www.bioinformatics.com.cn/plot).

van Galen and colleagues integrated single cell mutation detection and transcriptomes for 16 AML patients (35). Six types of AML cells resembled normal cell types along the differentiation axis from hematopoietic stem cell (HSC) to myeloid (HSC-like, progenitor-like, granulocyte-monocyte progenitor (GMP)-like, promonocyte-like, monocyte-like, and conventional dendritic cell (cDC)-like malignant cells) were classified (**Figure 1A**). Importantly, the relative abundances of these cell types were strikingly heterogeneous among tumors. Some AMLs consisted of major one or two cell types, while others contained a range of malignant cell types. Wu et al. used Microwell-seq and SMART-seq to analyze samples from 40 AML patients and identified a “cloud cluster” with no functional marker genes after compared with normal BM samples (36). Genetic network and correlation analysis showed that this “cloud cluster” resembles hematopoietic stem and progenitor cells (HSPCs). Gene expression profiling revealed upregulation of a spectrum of ribosomal protein (*RP*) genes in these AML progenitors. Despite of common features, AML progenitors could be divided into 16 sub-clusters and summarized into 4 main groups, including *RP* gene-High, neutrophil-like, monocyte-like, and myeloid cell-like. The number of cycling cells and activation of pathways were markedly different in some clusters, even within the same main group. Refractory-specific cluster cells overexpressed *MYC*, *SRC*, *RELA*, *MTOR* compared to non-refractory cluster cells in AML-M5 subtype (**Figure 2A**) (36).

Intratumoral heterogeneity has been dissected not only in same subtype based on AML morphology, but also in AML with same driving genetic lesion. *NPM1* is the most common mutated gene in AML, occurring in approximately 30% of adult AML. *NPM1* mutations result in the cytoplasmic localization of NPM1 (NPM1c) protein. In *NPM1* ^mut^ AML progenitors, 15 distinct clusters in 6 major group were identified distributed across the AML landscape, including early progenitors, myeloid progenitors, erythroid-like precursors, actively cycling blasts, differentiating myelo-/monoblasts and a microcluster with NK-like characteristics (**Figure 1B**). These early progenitors expressed *CD34*, *CD99*, *HOPX* and *EGFL7*, the host gene for *miR-126* (**Figure 2B**) (37). The distribution of these clusters evidently varied among *NPM1* ^mut^ patients. Some predominantly mapped to one or two groups, and others showed an extensive presentation across the whole landscape. Intrapatient cellular heterogeneity in t(8;21) AML has been explored at single cell level too (38). Combined with clinical immunophenotypic characterization, scRNA-seq analysis identified 5 distinct intrapatient leukemic cell clusters: CD34^+^CD117^dim^ blasts, CD34^+^CD117^bri^ blasts, CD34^+^CD117^bri^-S blasts, CD34^+^CD117^bri^-G2M blasts, and abnormal myeloid cells with partial maturation (AM) (**Figure 1C**). Transcriptomic profiling revealed well-defined gene signatures in these clusters, for example, cell migration and adhesion genes in CD34^+^CD117^dim^ blasts and cell cycle and DNA replication genes in CD34^+^CD117^bri^-S and CD34^+^CD117^bri^-G2M blasts (38). Similarly, combined whole-exome sequencing (WES) with scRNA-seq in longitudinal analysis of t(8;21) AML and *FLT3-ITD* AML revealed substantial heterogeneity both within and between blast cells of each patient and more heterogeneity among diagnosis-relapsed pairs (39). The most pronounced transcriptional variances were linked to large-scale copy number variations specific to each patient. Additionally, somatic variants, such as single nucleotide polymorphisms and small insertions and deletions contributed to further heterogeneity, highlighting distinct abundance and dynamics of AML clones unique to each patient (39). The key findings of some of these studies were summarized in **Table 1**.

**Table 1:** Summary of some studies of scRNA-seq in AML.

Key finding	Sample size	scRNA-seq platform	Suppl. techniques	Reference
1. identify 15 distinct cell types in healthy bone marrow 2. Reveal six types of AML malignant cell along the hierarchy of myeloid cell differentiation 3. High HSC/Prog-like signals had worse outcome than those with GMP-like signals 4. Monocyte-like cells potently inhibited T-cell activation (immunosuppressive)	16 AML (35 BM samples) 5 HDs	Seq-well	1. Targeted DNA seq 2. Sc genotyping by short- read seq 3. Sc genotyping by nanopore seq	(35)
1. Normal cell type: lymphoid, erythroid, and myeloid lineages (6 types of neutrophils) 2. Identify 20 cell clusters in AML and Type I (short survival) and Type II AML 3. Patients with ribosomal protein (RP) high progenitor cells had a low remission rate	40 AML 3 HDs	Micro-well Seq	Long-read single-cell targeted SMRT seq	(36)
1. A generalized inflammatory and senescence-associated response induced by chemotherapy 2. Heterogeneity within progenitor AML cells: OxPhos^high^ vs miR-126^high^OxPhos^low^ 3. miR-126^high^OxPhos^low^ LSCs are more quiescent with stemness, associated with refractory and relapse in *NPM1* mutant AML.	20 AML	10X Genomics	1. Immunophenotyping 2. NPM1 Mutation Finder, NPM1-MF	(37)
1. Three distinct leukemic cell populations identified: CD34^+^CD117^dim^ blasts, CD34^+^CD117^bri^ blasts, and abnormal myeloid cells with partial maturation (AM). 2.CD34^+^CD117^dim^ cells overexpress ell migration and adhesion genes, while CD34^+^CD117^bri^ cells overexpress cell cycle and DNA replication genes. 3. CD34^+^CD117^dim^ cells show higher LSC17 score compared to CD34^+^CD117^bri^ cells 4. A high proportion of CD34^+^CD117^dim^ cells in t(8;21) AML patients predicts inferior outcomes.	9 t(8;21)-AML	10X Genomics	Immunophenotyping	(38)
1. Pathway switch from AP1-regulated clone at diagnosis to mTOR-driven clone at relapse in *DNMT3A*/*FLT3-ITD* AML 2. Shared LSC signature between diagnosis and relapse in two ETO-AML cases 3. Tumor heterogeneity among patients with similar initiating mutations, also between each diagnosis-relapse pair	6 AML (diagnosis-relapse pair)	Single cell SORT-seq	1. WES 2. Fusion genes detection by RNA-seq	(39)
1. Identify18 clusters into 8 main cell populations 2. One LSC-like cluster with known LSC marker (CD34, CD96, CD133) and nontraditional LSC markers (CD38, CD46)	5 normal karyotype (NK) AML (M4/M5) 1 HD	10X Genomics	None	(40)
1. The fraction of progenitors is significantly higher in non-CR AML than CR AML, suggesting early hematopoiesis arrest in non-CR AML. 2. Distinct LSC markers uncovered in HSC-like cells from non-CR (CD9, CD82, CD123, IL1RAP)	13 AML (8 CR; 5 non-CR)	10X Genomics	None	(41)
1. CD99^+^CD49d^+^Galectin-1^+^CD52^+^ quiescent stem-like cells (QSCs) are involved in the chemoresistance and relapse of AML. 2. Interaction between QSCs and monocytes mediated by CD52-SIGLEC10 leads to immune suppression and poor outcomes. 3. LGALS1 is a promising target for refractory and relapsed AML.	10 AML (refractory and early relapsed)	10X Genomics	None	(42)
1. Inflammatory BM niche inhibits normal hematopoiesis, but not LT-HSCs and *NPM1* mutant leukemic cells. 2. Niche remodeling provides the competitive advantage of mutated cells over their normal and preleukemic counterparts, promoting leukemogenesis.	6 *NPM1*-mutant AML 4 HDs	10X Genomics	1. Immunophenotyping 2. Bulk RNA-seq 3. *NPM1* mutation identification by scRNA-seq	(43)

HDs: health donors; Suppl., Supplementary; WES, Whole exon sequencing; Sc, Single cell.

Taken together, scRNA-seq technology has significantly advanced our understanding of AML by uncovering a spectrum of distinct clusters of leukemic cells at different stage of differentiation trajectory. The representation of these clusters is markedly varied among AML patients.

## Characterizing leukemia stem cells

Differentiation block or maturation arrest is a key characteristic of AML disease, allowing AML blasts to proliferate continuously without undergoing the terminal differentiation and apoptosis process (44, 45). At the core of the blocked differentiation in AML are LSCs (46, 47). LSCs represent a subset of AML cells with a unique capability of initiating and maintaining a cellular hierarchy in AML (48). A large literature suggests that LSCs are responsible for the persistence of the disease and can be refractory to standard chemotherapy, resulting in relapse (47–50). Chemoresistance and relapse are the leading cause of AML-related deaths (51–53). scRNA‐seq technology has added value in LSC biology by unveiling novel surface makers, and distinct transcriptomics.

**Figure 3:**
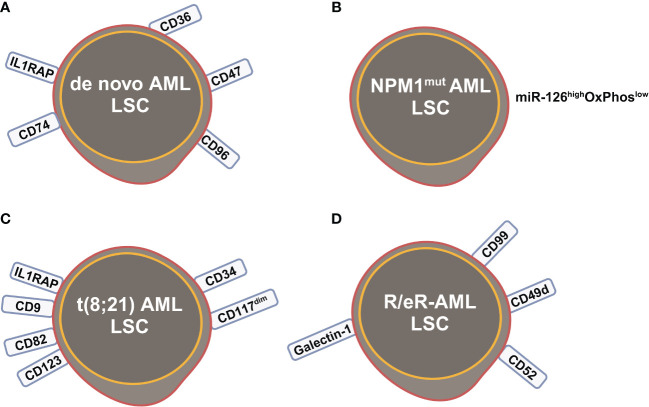
Leukemia stem cells (LSCs) identified by scRNA-seq studies in **(A)** *de novo* AML; **(B)** *NPM1* ^mut^ AML; **(C)** t(8;21) AML; **(D)** refractory and early relapsed (R/eR) AML. This figure was created with BioRender.com.

The primitive AML cell types identified in the study of van Galen et al. expressed established LSC markers, such as *CD96*, *CD47*, *IL1RAP* and *CD36*, and the additional candidate *CD74* (**Figures 2C**, **3A**) (35). CD96, a member of the Ig gene superfamily, is an LSC-specific marker in AML and is associated with dismal survival (54–57). CD74, also known as MHC HLA-DR gamma chain, plays an important role in AML cell survival in a network with LGALS3 (58). AML with more primitive LSCs inferring from higher HSC/Prog-like gene signature had significantly worse outcomes (35).

Integrated single cell transcriptomic data from all four patients described by Velten and colleagues (59), along with data from CD34^+^ BM cells of a healthy individual, revealed a subpopulation of quiescent immature HSC-like leukemic cells. These LSCs have increased expression of AP1 transcription factors (*FOS*, *JUN*, *FOSB*, *JUNB*, *JUND*) and decreased *MHC class II* expression (**Figure 2D**) (59). Interestingly, CD96, a known LSC marker mentioned earlier, was upregulated in only one patient, suggesting that LSC might be patient-specific too. In normal karyotype AML (FAB AML-M4/5), a presumable LSC cluster highly expressing *CD34*, *CD38*, *CD96*, *CD46*, *CD47*, CD82, *CD44* and *CD133* was uncovered (**Figure 2E**) (40). However, due to the small sample size in these two studies (40, 59), further validation of these findings in a larger cohort of AML patients is necessary to determine their general applicability. In *NPM1* ^mut^ AML, scRNA-seq provided a higher resolution and further anatomized the miR-126^high^ LSCs into dormant and cycling sub-compartment. Exhibiting overexpression in AML, miR-126 restrains cell cycle progression, inhibits differentiation, and enhances self-renewal of LSCs *in vivo* (60). The dormant miR-126^high^ LSCs had low expression of oxidative-phosphorylation (OxPhos) signatures and this miR-126^high^OxPhos^low^ population was enriched after chemotherapy in AML patient-derived xenografts (PDX) model (**Figure 3B**). A miR-126^high^ gene signature derived from these AML xenografts could identify a subset of chemotherapy-resilient LSCs enriched in refractory and relapsed AML. This signature also predicts poor survival in patients with *NPM1* ^mut^ (37). A subpopulation of CD34^+^CD117^dim^ cells were located at the earliest stage of differentiation as shown by single cell trajectory analysis of t(8;21) AML (**Figure 3C**). Longitudinal scRNA-seq at different disease stages revealed CD34^+^CD117^dim^ blasts expanded at post-relapse refractory stage after several cycles of chemotherapy (38). Importantly, higher percentage of CD34^+^CD117^dim^ cells in AML patent with t(8;21) was an indicator of poor prognosis (38). Chemoresistant HSC-like cells in non-CR AML were found to express more LSC markers, including *CD9*, *CD82*, *CD123* (*IL3RA*), and *IL1RAP* than those from CR AML (**Figure 2F**) (41). CD9, enriched in the CD34^+^CD38^-^ fraction of AML cells, is associated with chemoresistance (61, 62). However, the prognostic value of CD9 in AML remains elusive as contradictory results have been reported (62–64). CD82, also known as tetraspanin-27, is a member of the tetraspanin superfamily of cell surface proteins. CD82 plays multiple roles in promoting AML cell survival, adhesion, migration, resistance to Ara-C via activation of STAT5 pathway, PKCα and β1 integrin, N-cadherin (65, 66). CD123 has been well-studied in LSC biology, contributing to poor prognosis, high-risk, resistance to apoptosis and drug resistance of AML (67–69). CD123 serves a promising target for novel immunotherapies against AML and numerous clinical trials are currently ongoing (70–74).

Longitudinal scRNA-seq analyses of refractory and early relapsed AML (R/eR-AML) uncovered that, unlike proliferating stem/progenitor-like cells (PSPs), a distinct subpopulation identified as quiescent stem-like cells (QSCs) played a pivotal role in AML chemoresistance and led to adverse clinical outcomes. The QSCs had increased expression of *CD52* and *LGALS1* mRNA and a combination of cell surface markers: CD99^+^CD49d^+^CD52^+^Galectin-1^+^ (**Figure 3D**). Chemotherapy induced reprogram of PSPs to obtain a QSC-like expression pattern in refractory AML, leading to accumulation of QSCs. The presence of QSCs at diagnosis could be associated with chemoresistance, and these cells were further enriched in the residual AML cells of refractory patients (42).

In summary, scRNA-seq has unveiled significant heterogeneity within the AML LSC population. There isn’t a single uniform profile but rather distinct subpopulations of LSCs with varying gene expression patterns and functionalities. The identification of multiple LSC markers and diverse LSC gene expression patterns/signatures underscores the complexity and heterogeneity within these cell populations. Such diversity significantly influences their response to therapy and their refractory/relapse mechanisms.

## Scrutinizing bone marrow microenvironment

The BM microenvironment (niche) is a highly complex network, consisting of a cellular compartment, an extracellular matrix, and a liquid compartment (75). Various cell types including hematopoietic and nonhematopoietic cells such as BM mesenchymal stromal cells (BMSC), osteoblasts, osteoclasts, adipocytes, fibroblasts, BM endothelial cells (BMEC), and effector immune cells, inhabit and interact within the cellular compartment alongside the extracellular environment. The liquid compartment contains a mixture of growth factors, cytokines and chemokines (76, 77). Mounting evidence suggests that BM microenvironment can influence and regulate functions of HSCs, orchestrating hematopoiesis. The role of the BM microenvironment in supporting AML cell survival, fostering resistance to conventional chemotherapy and targeted treatments, and ultimately contributing to disease relapse has garnered growing interest (77–79). ScRNA-seq allows the identification and characterization of individual cell types within this BM microenvironment, offering a detailed understanding of the BM atlas and its interactions with AML cells.

scRNA-seq studies have been performed to define a cellular taxonomy of the mouse BM microenvironment and its perturbation by malignancy and stress (80–82). The initial comprehensive atlas of the mouse BM microenvironment identified 17 distinct cellular subsets, including BMSCs, osteolineage cells (OLCs), pericytes, BMECs, chondrocytes, and fibroblasts (80). Their putative functions and developmental relationships have been annotated too. Several scRNA-seq studies revealed novel insights into the interactions between AML cells and BM niche.

Leptin receptor (LepR) is a transmembrane receptor protein involved in responding to the hormone leptin. LepR^+^ cells are key components of the BM hematopoietic microenvironment, and involved in regulating hematopoiesis, bone formation and remodeling (83, 84). In the MLL-AF9 knockin leukemic model, LepR^+^ BMSCs were found to exhibit downregulation of key HSC niche factors like *ANGPT1*, which acts as an agonist for TEK receptors present on BMECs and HSCs. Additionally, factors promoting lymphoid or myeloid differentiation, as well as HSC homing to the bone marrow, were also observed to be downregulated (80). *SPP1*, an osteoblastic maturation marker that acts as an inhibitor of HSC pool size and proliferation, was noted to be upregulated in AML. In addition, osteogenic differentiation blockage in LepR^+^ BMSCs and OLCs could also be induced by leukemic cells, leading to remodeling and changes in bone composition. *WNT1-inducible-signaling pathway protein 2* (*WISP2*), known to inhibit MSCs ability to differentiate, was observed to be upregulated in all LepR+ BMSCs subsets as well as OLC progenitors, suggesting a compromised differentiation of LepR^+^ BMSCs and OLCs in AML (80). Under stress conditions such as chemotherapy, significant alterations in niche components occur, including the adipocytic skewing of perivascular cells regulated by the activation of adipogenesis-related pathways, as well as a widespread reduction in gene expression linked to the osteolineage (81). Consequently, the transcriptional remodeling of nice altered the normal hematopoiesis process. For example, downregulation of vascular Notch delta-like ligands (encoded by *DLL1* and *DLL4* genes) prematurely turned on a myeloid transcriptional program in hematopoietic stem cells (81).

Results from scRNA-seq in AML patients and healthy donors are consistent with these findings in mouse (43). In human BM from healthy donors, LepR^+^ BMSCs were identified as a key “communication hub”, playing central roles in in the homeostatic regulation of HSC and BM niche cells (43). Subclustering analysis identified 4 types of BMSC, ie, BMSC-0-3, with different level of *LepR* expression. The BMSC-0 population expressed highest level of *LepR* gene and *CXCL12*, *KITLG*, *ANGPT1*, and *IL7*. These genes encode critical HSPC regulatory factors. Accordingly, BMSC-0 are predicted to have the strongest interactions across HSPC subsets, particularly long-term (LT)-HSCs (43). In *NPM1* ^mut^ AML, BMSCs undergo inflammatory remodeling in which gene signatures reflecting inflammatory signaling, such as “TNFα signaling via NFκB”, “IL2–STAT signaling”, and “inflammatory response”, are among the most upregulated. The inflammatory remodeling disrupted the homeostasis of BMSC subclusters, resulting in a significant decrease in the BMSC-0 cluster, which was expected to have the strongest interaction with HSPCs, alongside a concurrent nearly seven-fold increase in BMSC-2. In all cases of *NPM1* ^mut^ AML, the BMSC-2 cluster consistently displayed a transcriptional profile characterized by the upregulation of genes associated with inflammation, as well as genes and signatures related to cell-extracellular matrix (ECM) remodeling. This indicates a significant expansion of an inflammatory subset, accompanied by a concurrent decline in the BMSC subset expected to sustain the normal maintenance of HSPCs (43). Collectively, LepR^+^ BMSCs in AML undergo remodeling due to inflammatory activation. This results in a significant expansion of an inflammatory subset (BMSC-2) alongside a simultaneous reduction in the BMSC subset expected to sustain the maintenance of normal HSPCs (BMSC-0).

A profound shift in cell-cell interaction between hematopoietic BM niche and HSPCs has been found in AML patients when compared to healthy donors (85). At the time of diagnosis, there was a notable increase in the abundance of the most primitive myeloid progenitor population, HSC/multipotent progenitor cell (MPP). Following treatment and achieving remission, these proportions reverted to levels comparable to those observed in a healthy state (85). However, during relapse, there was a resurgence in the enrichment of myeloid progenitors. Predictive analyses unveiled interactions between HSPCs and other BM cell types, revealing a notable expansion of interactions in AML that promote HSPC-cell adhesion, immunosuppression, and cytokine signaling. Specifically in AML, integrin β1 (ITGB1, CD29) was predicted to form interactions with a broader spectrum of ligands, which promoted adhesion and survival of HSC and MPP (85). The transforming growth factor-β (TGF-β) signaling pathway plays a crucial role in regulating various cellular processes, including normal hematopoiesis, and its dysregulation has been implicated in AML (86, 87). The interaction between TGFβ1 and TGFBR2 was predicted to be widespread in AML when compared to healthy controls (85). Increased expression or secretion of TGFβ1 inhibits normal HSC proliferation and is linked to the quiescence state of LSC in AML (85–87). Additionally, ECM and cytokine production have been observed in AML too (85). However, caution should be exercised regarding these predicted interaction changes due to the lack of rigorously experimental validation in this study (85).

Taken together, these findings from scRNA-seq in the context of AML suggest that malignant cells can affect normal hematopoiesis by remodeling in BM microenvironment composition and alter the regulation of HSC niche factors in the stroma. This compromises the BM microenvironment to be less conducive towards normal hematopoietic cell production, but confers competitive advantage to AML cells, such as adhesion, survival, quiescence for the AML cells.

## Divulging the immune system

Immune surveillance mechanisms, comprising adaptive and innate immune systems, are natural protectors in preventing hematological malignancies (88). A large body of pre-clinical and clinical studies indicates the substantial contribution of compromised immune surveillance mechanisms to the establishment of preleukemic states and their progression toward AML (89–92). Recent studies using scRNA-seq provide comprehensive view of immune escape strategies employed by AML cells to evade immune recognition, as well as AML cell-induced modifications of various immune cell populations, including T cells, natural killer (NK) cells, dendritic cells, and myeloid-derived suppressor cells (93–97).

**Figure 4:**
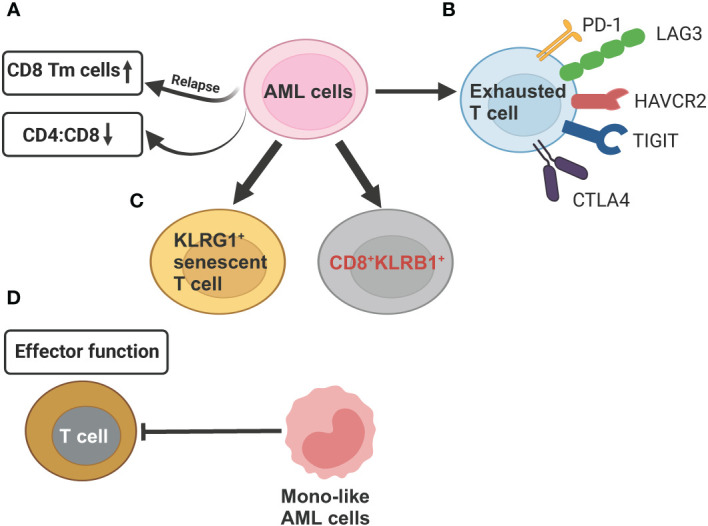
Schematic illustration summarizing the compromised T cell functions mechanisms exerted by AML cells revealed by scRNA-seq studies. **(A)** AML cells can reduce CD4:CD8 ratio as compared to healthy controls. In relapsed AML, there is an increase in CD8^+^ memory T cells (Tm) when compared to newly diagnosed AML. **(B)** AML cells induce T-cell exhaustion by increasing expression of inhibitory T-cell receptors (PD-1, LAG3, HAVCR2, TGIT, CTLA4. **(C)** In relapsed AML, the expansion of KLRG1^+^ senescent T cell and CD8^+^KLRB1^+^ T cells contribute to chemoresistance. **(D)** Differentiated monocyte-like AML cells hamper effector T cell function. This figure was created with BioRender.com.

The development of AML leads to profound alterations in the lymphoid lineage, including a significant reduction in the proportion of common lymphoid progenitors (CLPs) and their offspring, such as pre-B cells, mature B cells, as well as, to a lesser extent, CD4^+^ and CD8^+^ naive T cells and CD4^+^ memory T cells (35). Although both AML and healthy control samples have the same two T cell subsets, naive T cells and cytotoxic T cells (CTLs), and a related population of NK cells, their proportion and function differ. AML samples have comparatively fewer T cells and CTLs but relatively more regulatory T cells (T-regs) compared to healthy controls. Overall, these findings underscore long-term damage to the adaptive immune system and an immunosuppressive BM niche of AML (35). It has been recently showed that differentiated monocyte-like AML cells exhibit features of classical and non-classical monocytes, but lack cytotoxic signature genes (35). These differentiated monocyte-like AML cells can inhibit T cell activity, contributing compromised immune tumor surveillance in AML (35). Another observation that aligns with the immune suppressive state of AML disease is the enrichment of CD8+ memory T cells at relapse compared to diagnosis (94) (**Figure 4A**). This suggests that while cytotoxic cell numbers recover during remission, they are functionally ineffective to execute anti-AML immune responses, potentially contributing to relapse.

Several scRNA-seq studies, paired with single-cell T cell receptor (TCR) sequencing on T cell, have defined high-resolution atlas of different T cell populations with distinct functions in AML. In general, the clonotype size, denoted by the count of cells expressing identical TCR sequences, is highest in refractory/relapsed AML, followed by newly diagnosed AML and healthy donors, revealing that T cells are more clonal in the AML microenvironment (93, 94, 97). Five T cell phenotypes, including 2 conventional (CD4^+^, CD8^+^) and 3 unconventional, such as gamma-delta (γδ) cells, mucosal associated invariant T-cells (MAIT) cells, and all other (unconv T) cells were identified in AML BM samples. AML had lower CD4:CD8 ratio in BMs than that in healthy BMs (93) (**Figure 4A**). CD8+ T cells in AML could be further divided into 16 clusters, forming 6 major types, including naive, memory, effector memory, CTL, MAIT, and exhausted (94). The percentage of exhausted population was lowest in AML compared to other 20 types of tumors using the same exhaustion definition (98), which involves coexpression of *PDCD1*, *TOX*, *CXCL13*, *TIGIT*, *CTLA4*, *TNFRSF9*, *HAVCR2*, and *LAG3* (94) (**Figure 4B**). This finding of a lower abundance of CD8^+^ T cells expressing canonical immune inhibitory–related markers in AML has important clinical significance, partially explaining the limited efficacy of immune checkpoint inhibitors observed in AML patients, in contrast to remarkable responses seen in some solid tumors. The CD8^+^ effector T cells are markedly different between newly diagnosed and refractory/relapsed AML (**Figures 4C, D**). Refractory/relapsed AML shows relatively higher abundance of clusters enriched with expression of the inhibitory receptor *KLRG1*, a marker of antigen-experienced and senescent cells (99, 100), while newly diagnosed AML displays relative higher percentage of clusters expressing cell-migratory receptor *CXCR4* and AP-1 transcription factor *FOSB* (94). Thus, these heterogeneous clusters of CD8^+^ effector T cells with different cellular states might contribute to refractory/relapsed disease.

In a study of analyzing normal karyotype AML (M4/M5), seven T cell clusters were discovered, including 4 clusters of CD8^+^, 2 clusters of CD4^+^ cells NFE2 cluster, CD4^-^CD8^-^ cluster, based on their gene expression characteristics. MAIT cells express a semi-invariant TCR called the TCRα7.2, which recognizes microbial-derived metabolites presented by the MHC-related protein 1 (MR1) (101, 102). The cluster, predominantly composed of MAIT cells (97), showed a higher proportion in refractory and relapsed cases compared to the other samples, implying a potential role of MAIT cells in the pathogenesis and disease progression of normal karyotype AML (97). Another study of PB mononuclear cell (PBMC) samples in AML patients also found predominant MAIT cells in one of the CD8^+^ T cell clusters (95). However, the clinical significance of MAIT cells was not explored in this study (95). Five subclusters of CD4^+^ T cells and four subclusters of CD8^+^ T cells were also defined based on transcriptomic signatures. While the proportion of cytotoxic CD4^+^ effector T cells varied among AML patients, it was generally lower compared to healthy donors. This suggests that the reduced presence of these immune cells contributes to the progression of AML (95).

NK cells are a type of cytotoxic innate immune cells that produce inflammatory cytokines and chemokines (103). They play a crucial role in the immune response by lysing infected and cancer cells, including AML (104). NK cells are generally divided into 2 classic subsets, cytokine-producing CD56^dim^ and cytolytic CD56^bright^ NK cells (105). Recent single-cell transcriptomics study analyzing NK cells in PB from healthy donors revealed six subtypes of NK cells, including 3 well-defined subsets (CD56^bright^CD16^−^, CD56^dim^CD16^+^CD57^−^, and CD56^dim^CD16^+^CD57^+^) and 3 novel subsets (type I interferon–responding NK cells, cytokine-induced memory-like NK cells, and population with low-ribosomal expression) (96).

Comparative scRNA-seq studies have been performed on NK cells from healthy donors and AML BM samples to better understand NK cell dysfunction in AML. Crinier et al. identified three NK cell populations common in 8 healthy donors based on gene signature analysis. Among them, two populations resembled CD56^dim^CD16^-^ and CD56^bright^CD16^-^ NK cells, while the third cluster was constituted of a CD56^bright^ tissue-resident NK cell population that resides in the spleen and is absent from the blood (106). In contrast, NK cells in AML patients were profoundly heterogeneous and exhibited patient-specific features. There was no distinct NK cell subset shared among AML patients, suggesting that the NK cells were impacted by AML cells in an individual fashion (106). Transcriptomic profiling demonstrated that NK cells from AML patients had higher expression of interferon-induced genes, while NK cells from healthy donors expressed higher level of NK cell effector molecules. CD160 is an important human NK cell activating receptor and its decreased expression is associated impaired NK cell function. AML patients with lower expression of CD160 have poorer survival than those with higher level of CD160 (106). In PBMC samples, the number of NK cells was lower in AML patients than that in healthy donors, indicating the AML-induced suppressive circumstance (95). However, they shared same NK cell expression makers and formed a single functional subtype. Differential gene expression analysis revealed downregulation of 10 transcription factors involved in homeostatic NK cell proliferation and survival, such as *CEBPD*, *KLF3*, *KLF2*, *USF2*, and *FOXP1*, in AML patients (95). A unique subset of NK cells characterized by the CD56^bright^CD16^hi^ phenotype, particularly in the hypomaturation stage, is prevalent in AML. The hypomaturation stage of these NK cells has been linked to decreased overall survival (OS) and event-free survival (EFS) in AML patients (107). In contrast to other NK cell populations, the CD56^bright^CD16^hi^ cells displayed distinct shifts in both phenotype and function, marked by remarkably low scores for activating and inhibitory receptors (108). Current NK cell-based therapies primarily aim to enhance NK cell activation and longevity, often overlooking the heterogeneity among cancer types and the suppressive influence of the tumor cells and tumor microenvironment on NK cell cytotoxic functions. The new knowledge of NK cell dysfunction gained from extensive scRNA-seq research is crucial for shaping future therapeutic strategies for AML and other cancers in general.

## Expanding therapeutic opportunities

With a deeper understanding of the genetic and transcriptomic landscape of AML at the single-cell level, new cell type-specific targets can be identified and more tailored and effective treatments, such as targeted therapies or immunotherapies, can be developed.

As mentioned earlier, *CD52*, *LGALS1* and *CD47* are significantly elevated in QSCs cells compared to other cellular states (42). Additionally, LGALS1 expression is also found to be significantly increased in chemo-residual QSCs in refractory AML patients and in daunorubicin-resistant leukemia cell lines compared to sensitive cells (109). Furthermore, scRNA-seq comparison of CD34^+^ BM cells at diagnosis from CR vs non-CR AML patients revealed that a cluster of GMP cells characterized by CRIP1^high^LGALS1^high^S100As^high^ was significantly enriched in non-CR samples and associated with poor prognosis of AML (110). AML patients with higher expression of LGALS1 had a worse OS and EFS that those with lower LGALS1 expression in the TCGA cohort (42). Overall, these data demonstrate that LGALS1 may mediate the chemoresistance and represent a novel therapeutic target for resensitizing QSC LSCs to chemotherapy. The LGALS1 inhibitor, OTX008, has been shown to enhance the chemotherapy in AML cell lines, primary AML cells, cell lines, and to eliminate the chemoresistant QSCs in AML PDX models (42). Other markers specifically expressed on QSCs, such as CD52 and CD47 are potential targets for the development of small molecule inhibitors or immunotherapies.

Chimeric Antigen Receptor T-cell (CAR-T) therapy, involves genetically engineering T cells to express chimeric antigen receptors, enabling them to recognize and eliminate tumor cells (111). However, only limited efficacy of CAR-T therapy targeting CD33, CD123 in AML have been observed in clinical trials (112). The widespread occurrence of adverse events resulted from CAR-T therapies is the “on-target, off-tumor toxicity”, which arises in patients who have target antigen expressed on both tumors and healthy tissues (113). Unfortunately, frequently targeted antigens in AML CAR-T therapy, such as CD33 and CD123, found in around 80–90% and 70–80% of AML patients, respectively, are also detected on HSCs and normal myeloid progenitor cells (114). This lack of target specificity can lead to unintended toxicity, prolonged severe myelosuppression, and dependence on transfusions. scRNA-seq is a powerful tool for comparing the expression levels of antigens in malignant cells and non-malignant cells from a broad range of healthy tissues.

The expression patterns of common AML-related target antigens, including *CD33*, *CD123*, and *CLEC12A* (*CLL-1*, *CD371*), were investigated in normal tissues and organs (115). Targeting CD33, CD123, and CLEC12A primarily affected CD14^+^ monocytes, CD16^+^ monocytes, and DC populations, with minimal impact on other hematopoietic lineages, such as B lineages, T lymphocytes, and NK cells. Notably, all these genes exhibited expression at the mRNA level in partial on HSPCs. A major concern arises from the higher frequency of *CLEC12A* in platelets at the mRNA level, and the abundant presence of *CD123* in multiple pancreatic cell types, as well as ECs across various organs, including cardiac, lung, skin, liver, and urinary bladder (115). *CD123* was also expressed in a small number of cardiac fibroblasts, aortic fibroblasts/smooth muscle cells/mesenchymal stem cells (MSCs), and lung epithelial cells (115). Thus, CD123 CAR-T can inadvertently injure these healthy cells, causing damage to endothelial cell and hematopoietic toxicity, including prolonged myelosuppression. Meanwhile, CD33 CAR-T treatment could eradicate skin Langerhans cells, potentially compromising the skin’s defense against pathogenic microorganisms (115). Therefore, the widespread of “on-target, off-tumor toxicity” associated with these CAR-T therapies poses major limitations to their application in treating AML. The selection of the right target antigens for CAR-T immunotherapy in AML remains a significant challenge.

In attempt to search for novel CAR-T targets with minimal or none of “on-target, off-tumor toxicity” in AML, Gottschlich and colleagues utilized a comprehensive RNA-sequencing dataset comprising over 500,000 single cells from 15 AML patients and 9 healthy individuals (116). After a serial of stepwise filtering, colony-stimulating factor 1 receptor (CSF1R) and CD86 were identified as novel target antigens for CAR-T cell therapy in AML. The expressions of *CSF1R* and *CD86* were higher on malignant HSC-like and HSPC-like cells than on healthy controls. Furthermore, *CSF1R* and *CD86* were lack on T cells and had minimal expression on nine organs in healthy controls (116). Functional validation of these CAR-T cells demonstrated robust efficacy in both *in vitro* and *in vivo* AML models, with minimal off-target toxicity to relevant healthy tissues (116). These findings provide a compelling basis for advancing these CAR-T cells into further clinical development. The high-resolution, single-cell expression analysis offers an innovative strategy for identifying new CAR-T targets in AML. The ongoing clinical trials of these new CAR-T therapies give an opportunity for combating AML and improved outcomes for patients, especial for refractory and relapsed cases in the future.

## Conclusion and perspective

The past few years have witnessed substantial progress in scRNA-seq study of AML. These advances signify the cellular heterogeneity within the AML ecosystem, a complexity previously overlooked in bulk RNA-seq analyses. The insights gained from scRNA-seq greatly enhance our understanding of LSC, compromised immune cells, altered BM microenvironment, and mechanisms of resistance to chemotherapy and relapse. Furthermore, they pave the way for new therapeutic avenues, such as the development of truly AML-specific immunotherapies and small molecular inhibitors for unique LSC subpopulation.

Despite these advancements, certain challenges and unresolved issues persist in the application of scRNA-seq in AML. Spatial transcriptomics technologies have emerged as powerful instruments in cancer research, providing valuable insights into the spatial organization of gene expression within intact tissue sections in the original physiological context (117, 118). However, current scRNA-seq methods cannot provide spatial information, leading to a loss of spatial dimension regarding how different cell types and subpopulations interact directly within the BM microenvironment. While this is not a concern in solid tumor samples, it is not applicable to formalin fixed paraffin embedded (FFPE) BM samples because the harsh decalcification procedure can result in severe degradation of RNA (119). A technical breakthrough is required to enable the utilization of FFPE BM samples in conjunction with spatial transcriptomics and single-cell technologies. This advancement would facilitate the construction of 3D spatial maps of gene expression, providing a visual depiction of interactions among AML cells, including LSCs, immune cells, and stromal components, within their natural BM environments. Another major challenge is the reproducibility and comparability of results across studies due to the different scRNA-seq platforms, variations in sequence depth and bioinformatic pipelines applied (120). Hence, future scRNA-seq studies could be enhanced through the standardization of protocols and methodologies, alongside the creation of robust computational tools and analytical frameworks, complemented by artificial intelligence technology.

Furthermore, there is a need for scRNA-seq in combination with other omics technologies, such as genomics, epigenomics, proteomics and metabolomics to provide a more holistic view of gene mutations, methylation, acetylation, chromatin remodeling, protein abundance and substrates and products of metabolism (121–126). For example, AML is known for its cellular heterogeneity, with different subpopulations of AML cells exhibiting diverse gene expression profiles and epigenetic states. Single-cell ATAC-seq (Assay for Transposase-Accessible Chromatin with high-throughput sequencing) enable the genome-wide mapping of accessible chromatin regions at the single-cell level (127). scRNA-seq combined with single-cell epigenomics techniques, such as scATAC-seq, can provide simultaneous profiling of gene expression and chromatin accessibility in rare AML populations like LSC, delineating epigenetic dysregulation and transcriptional regulatory networks. Single-cell proteomics techniques, such as mass cytometry (CyTOF) (128–130) or single-cell proteomic assays (131, 132), complement scRNA-seq by profiling the expression levels of proteins at the single-cell level. Likewise, integrating scRNA-seq with single-cell proteomics enables comprehensive assessment on how a single gene’s protein levels track with its mRNA levels across individual AML cells, given the fact that genome-wide correlation between expression levels of mRNA and protein on bulk RNA-seq and proteomic studies are around 40% (133). Single-cell metabolomics using mass spectrometry allows for the simultaneous detection of a wide range of metabolites from individual cells (134, 135), Integration of scRNA-seq with single-cell metabolomics facilitates measurement of metabolite levels associated with key metabolic pathways, such as glycolysis, tricarboxylic acid cycle, and amino acid metabolism (136, 137), capturing both transcriptional signatures and metabolic profiles, within individual AML cells. CRISPR/Cas9 technology enables the targeted manipulation of specific genes in a controlled manner (138). By combining scRNA-seq with CRISPR/Cas9, like Perturb-seq (139) and Cas13 RNA Perturb-seq (CaRPool-seq) (140), researchers can systematically perturb potentially important oncogenes and assess the effects on the transcriptome on single cell level (141). This approach aids in pinpointing crucial oncogenic drivers involved in both the onset and advancement of AML, thereby streamlining the development of targeted therapeutic approaches designed to eliminate LSC populations responsible for disease recurrence.

The established prognosis factors used in clinic decision are age, cytogenetic abnormalities, molecular mutations (e.g., *FLT3*, *NPM1*, *CEBPA*, *DNMT3A*, *IDH1*, *IDH2*, *TP53*), and the new 2022 edition of European LeukemiaNet (ELN) risk classification (Favorable prognosis, Intermediate prognosis and Adverse prognosis) based on the genetic alterations (8). One major limitation of current risk stratification is primarily derived from clinical studies comprising younger, fit patients with *de novo* AML who received intensive chemotherapy. However, the average age at diagnosis for adult AML is 68 years. Older adult AML patients often receive lower-intensity therapy instead of intensive therapy (142). Therefore, this limitation significantly restricts the applicability of these guidelines. scRNA-seq has the capability to capture rare cell subsets, such as AML LSC or therapy-resistant clones, which may have prognostic implications. Importantly, the application of scRNA-seq in prognosis is applicable to both young and old patients, as well as to patients who have received either intensive or low-intensity chemotherapy, targeted therapies, or immunotherapies. Hence, incorporating scRNA-seq data with established risk factors, such as age, cytogenetic abnormalities, and mutation status, could improve prognostic accuracy.

Furthermore, scRNA-seq can be leveraged to monitor the dynamic changes in the AML cell and immune cell populations during treatment, enabling real-time tracking of treatment response and the emergence of resistant clones. These data derived from scRNA-seq can be used to identify transcriptional signatures associated with drug sensitivity or resistance in AML cells. The signatures may serve as predictive biomarkers for treatment response, which can guide personalized treatment adjustments, potentially minimizing the risk of relapse and improving patient survival. However, the clinical implementation of scRNA-seq in AML faces several limitations and challenges. One of the primary concerns is the cost of scRNA-seq, which can be prohibitively expensive for routine clinical use, particularly with high volume of samples. Secondly, the bioinformatic analysis of scRNA-seq data is complex and requires fair amount of computational resources and dedicated bioinformaticians, which may not be readily available in some resource-limited hospitals.

Despite these limitations, the potential advantages of scRNA-seq in improving AML diagnosis, prognosis, and treatment are indisputable. Moving forward, we provide our view on the trajectory towards clinical application of scRNA-seq. As a relatively young, but fast-evolving field of single-cell field, ongoing advancements in sequencing technologies, bioinformatics tools, and protocol optimization are driving down the cost of scRNA-seq and improving its scalability. We foresee the sequencing costs continue to decrease and technologies become more user-friendly, the implementation of scRNA-seq in clinical settings is expected to increase. Clinical validation studies are essential for establishing the clinical utility of scRNA-seq in AML diagnosis, prognosis, and treatment. Larger cohorts of AML patients and prospective, longitudinal studies are needed to determine the clinical utility of scRNA-seq in prognosis assessment and personalized medicine for AML patients. Regulatory approval and standardization of scRNA-seq protocols are critical steps towards its widespread adoption in clinical practice. With continued innovation, cost reduction, and validation in clinical trials, we expect scRNA-seq holds great promise for clinic use in management of AML patients in the near future.

Collectively, the perspective for integrating scRNA-seq into basic and translational research in AML is optimistic. The new insights uncovered by the widespread use of scRNA-seq are crucial for the development of novel therapies, especially for refractory/relapsed AML and significantly improve the survival rate of AML patients.

## Author contributions

JZ: Conceptualization, Formal analysis, Funding acquisition, Writing – original draft, Writing – review & editing, Resources. W-JC: Writing – original draft, Writing – review & editing, Funding acquisition, Resources.

## Glossary

**Table:**

*AML*	Acute myeloid leukemia
*ATAC-seq*	Assay for Transposase-Accessible Chromatin with high-throughput sequencing
*B-ALL*	B cell acute lymphoblastic leukemia
*BM*	Bone marrow
*BMEC*	BM endothelial cells
*BMSCs*	bone marrow mesenchymal stromal cells
*CaRPool-seq*	Cas13 RNA Perturb-seq
*CAR-T*	Chimeric antigen receptor T cell
*cDC*	Conventional dendritic cell
*CSF1R*	colony-stimulating factor 1 receptor
*CLPs*	common lymphoid progenitors
*CTLs*	cytotoxic T cells
*DLBCL*	Diffuse large B cell lymphoma
*FACS*	Fluorescence-activated cell sorting
*FFPE*	Formalin-fixed paraffin-embedded
FLT3	FMS-like tyrosine kinase 3
*GMP*	Granulocyte macrophage progenitors
*HSC*	hematopoietic stem cell
*HSPC*	hematopoietic stem and progenitor cell
*IDH1*	isocitrate dehydrogenase 1
*LepR*	Leptin receptor
*LSCs*	Leukemia stem cells
*LT-HSCs*	long-term hematopoietic stem cells
*MM*	multiple myeloma
*MPP*	Multipotent progenitor cell
*MSCs*	Mesenchymal stem cells
*NGS*	Next-generation sequencing
*NK cells*	Natural killer cells
*NPM1*	Nucleophosmin 1
*OLC*	Osteolineage cells
*OxPhos*	Oxidative-phosphorylation
*OS*	Overall survival
*PBMC*	Peripheral blood mononuclear cell
*PD-1*	Programmed cell death protein 1
*PDX*	Patient-derived xenografts
*PFS*	Progression-free survival
*QSCs*	Quiescent stem-like cells
*RNA-seq*	RNA sequencing
*RP*	Ribosomal protein
*sc*	Single-cell
*scRNA-Seq*	Single-cell RNA sequencing
*SMART-seq*	Switching mechanism at the 5’
*SORT-seq*	FACS sorting and robot-assisted transcriptome sequencing
*TCR*	T-cell receptor
*Tm*	Memory T cells
*T-regs*	Regulatory T cells
*WES*	Whole exome sequencing
*WGS*	Whole genome sequencing.
